# Single-photon non-linear optics with a quantum dot in a waveguide

**DOI:** 10.1038/ncomms9655

**Published:** 2015-10-23

**Authors:** A. Javadi, I. Söllner, M. Arcari, S. Lindskov Hansen, L. Midolo, S. Mahmoodian, G Kiršanskė, T. Pregnolato, E. H. Lee, J. D. Song, S. Stobbe, P. Lodahl

**Affiliations:** 1 Niels Bohr Institute, University of Copenhagen, Blegdamsvej 17, DK-2100 Copenhagen, Denmark; 2 Center for Opto-Electronic Convergence Systems, Korea Institute of Science and Technology, Seoul 136-791, Korea

## Abstract

Strong non-linear interactions between photons enable logic operations for both classical and quantum-information technology. Unfortunately, non-linear interactions are usually feeble and therefore all-optical logic gates tend to be inefficient. A quantum emitter deterministically coupled to a propagating mode fundamentally changes the situation, since each photon inevitably interacts with the emitter, and highly correlated many-photon states may be created. Here we show that a single quantum dot in a photonic-crystal waveguide can be used as a giant non-linearity sensitive at the single-photon level. The non-linear response is revealed from the intensity and quantum statistics of the scattered photons, and contains contributions from an entangled photon–photon bound state. The quantum non-linearity will find immediate applications for deterministic Bell-state measurements and single-photon transistors and paves the way to scalable waveguide-based photonic quantum-computing architectures.

## Abstract (web summary)

Interacting light beams are required for all-optical information processing, but such nonlinear effects are tiny at the single-photon level. Here, the authors show that a single quantum dot in a photonic-crystal waveguide enables the necessary giant optical nonlinearity.

## Graphical abstract

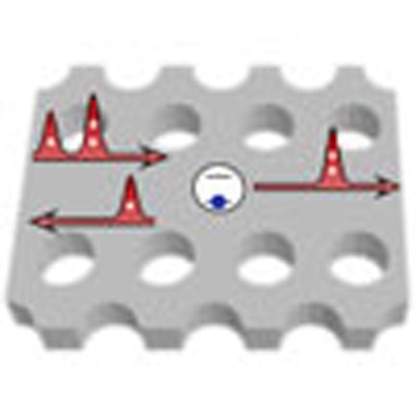

The access to an efficient optical non-linearity enables the processing of quantum information stored in light and generation of exotic states of light^1^,^2^,^3^,^4^,^5^. A non-linearity capable of operating down to the ultimate level of single photons has been long sought after, as it opens new avenues for photonic quantum-information architectures^6^,^7^, and enables efficient Bell-state analysers^8^ and single-photon transistors^9^. Moreover, such an efficient non-linearity could have extensive applications in optical signal processing^10^ and improve linear-optics quantum-information architectures^11^. Photonic nanostructures provide a route to overcoming these limitations since light and matter can be deterministically interfaced. One approach to photon non-linearities exploits the anharmonic spectrum of a cavity polariton, which has been experimentally demonstrated both with atoms^12^ and quantum dots^13^,^14^,^15^,^16^,^17^. An alternative approach uses the intrinsic non-linearity of a quantum emitter deterministically coupled to a single photonic mode (a ‘one-dimensional (1D)atom'); such a coupling was recently achieved with single quantum dots in photonic-crystal waveguides^18^. Theoretical studies have predicted that intricate photon–photon^3^ and photon-emitter^19^ entanglement (so-called bound states) may be induced by deterministic few-photon scattering. So far a quantum-emitter non-linearity has been observed at microwave frequencies with superconducting qubits^20^, at optical frequencies with single atoms in cavities^21^,^22^,^23^ and with single molecules in dielectric nanoguides^24^.

A quantum dot in a photonic waveguide is a particularly attractive approach to quantum non-linear optics since it can be naturally incorporated in integrated photonic circuits. For quantum dots in photonic cavities^14^,^15^ the precise tuning of the emitter to the cavity resonance is required. Moreover, the efficient outcoupling of photons from a high-Q photonic-crystal cavity can be challenging due to the complex mode structure of nanophotonic cavities, and coupling of PhC cavities to in-plane propagating modes compromises the Q-factor of the cavity. The very wide coupling bandwidth in photonic-crystal waveguides^25^ implies that scalable quantum architectures may be envisioned with significantly less experimental overhead, since only the quantum dot needs to be tuned. Also the planar propagation of the light in a waveguide enables a quantum-photonic chip, where all the required operations can be done on the same chip.

A quantum dot in a photonic-crystal waveguide constitutes the paradigmatic example of a 1D artificial atom where light-matter interaction is fundamentally different from that in 3D. For instance, dipole-induced single-photon interference may be studied, which can be considered a precursor to the experimental demonstration of single-photon non-linearity and photon–photon bound states. Furthermore, an anomalous radiative Lamb shift or infinitely ranging dipole–dipole interaction have been been predicted^5^.

The present work reports on single-photon non-linear optics in a waveguide-based scalable solid-state platform. We design a photonic circuit that allows us to probe single quantum dots, efficiently coupled to photonic-crystal waveguides. We show that a single quantum dot can modify the transmission of the waveguide by up to 30%. Our experimental results show that the transmission is highly non-linear and the non-linearity operates at the single-photon level. The critical average number of photons per lifetime of the emitter is 0.81. We also show that the statistics of the transmitted photons is bunched, which reveals the quantum nature of the non-linearity.

## Results

### Operational principle and sample structure

**Figure 1:**
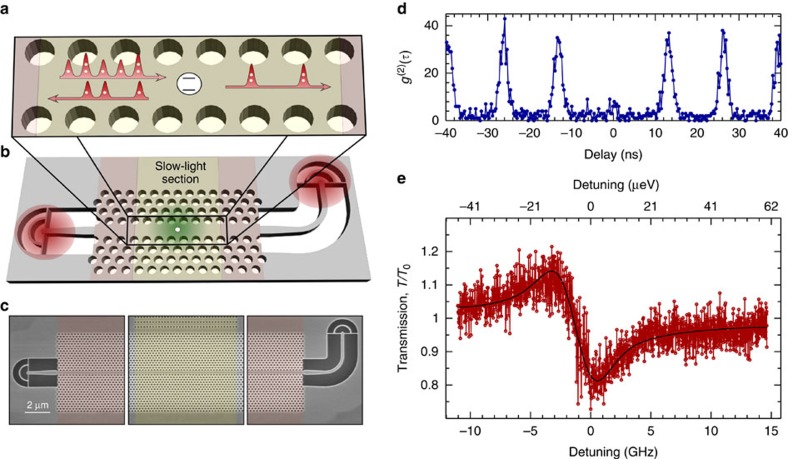
Resonant spectroscopy on a quantum dot in a photonic-crystal waveguide. (**a**) Operational principle of resonant scattering in a photonic-crystal waveguide. Single-photon components of the incoming light are reflected by the quantum dot while two- and more-photon components are preferentially transmitted. (**b**) Illustration and (**c**) scanning electron micrograph of the sample. A quantum dot (white circle) in the central part of the slow-light section is excited by launching light through one grating and detecting light from the other grating. The red areas indicate the size of the excitation and collection areas. The green area is the illumination region of the repump laser, see Supplementarty Information. (**d**) Autocorrelation function of the photons emitted from the target quantum dot. The central peak is highly suppressed, which proves the single-photon character of the system. The area under the suppressed peak is about 30% of the adjacent peaks. (**e**) Resonant transmission spectrum recorded by scanning a narrow-band continuous-wave laser through the resonance of a quantum dot in the case of weak excitation. The power on the sample was 50 pW, which is far below the critical power of 1.9 nW. The solid black line is a model of the experimental data of the Fano resonance, see Supplementarty Information for details of the model.

The operational principle of the quantum-dot non-linearity is outlined in Fig. 1a: by scattering a weak resonant laser on a single quantum dot in the photonic-crystal waveguide, the single-photon component is reflected while two- and higher-photon components have an increased probability of being transmitted. The layout and scanning electron micrograph of the sample are shown in Fig. 1b,c, respectively. The sample consists of a central slow-light waveguide section (slow-down factor *n*_g_∼30) terminated on each side by two waveguide sections (*n*_g_=5) and coupled through suspended waveguides to two gratings, which direct the emission vertically out of the planar structure. One grating is used for launching light into the waveguide and the other for extracting the transmitted light. Furthermore, the quantum dot is exposed to a repump laser to prepare and stabilize the initial state of the emitter before the scattering process. The detailed description of the sample and experimental procedure is presented in Supplementary Note 1 and 2, and Supplementary Figs 1 and 2. We choose a quantum dot line that has a wavelength of ∼914 nm for this experiment, see Supplementary Note 3 and Supplementary Fig. 3 for the emission spectrum of the sample. We verify that a single quantum dot is probed by recording the autocorrelation function of the emitted photons under pulsed excitation. Figure 1d shows the autocorrelation function versus time delay, while exciting close to the saturation power of the quantum dot. The antibunching of the photons observed around zero time delay proves the single-photon nature of the emission, see the Supplementary Note 4 and Supplementary Fig. 4 for the details of the measurement and the analysis. Figure 1e shows an example of the transmission spectrum recorded when scanning a narrow-linewidth laser through the resonance feature of a quantum dot coupled to a photonic-crystal waveguide. These measurements are performed at low excitation powers in the coherent-scattering regime where the incoming and outgoing fields maintain a fixed phase relation. Residual reflections from the waveguide ends imply that weak Fabry–Perot resonances form, which lead to a characteristic Fano line shape^26^,^27^ of ∼30% modulation. The peak (dip) of the transmission spectrum corresponds to dipole-induced transparency (reflection) resulting from single-photon interference. The size of the modulation is determined by the *β*-factor, which quantifies the coupling efficiency, as well as the spectral diffusion and blinking of the quantum-dot line due to charge or spin noise^28^, and residual broadening of the zero-phonon line, for example, due to carrier-phonon interactions^29^. Spectral diffusion and blinking processes can be strongly reduced by implementing electrical gates on the quantum-dot samples^28^ and can be further suppressed through active stabilization.

### Non-linear transmission of the waveguide

**Figure 2:**
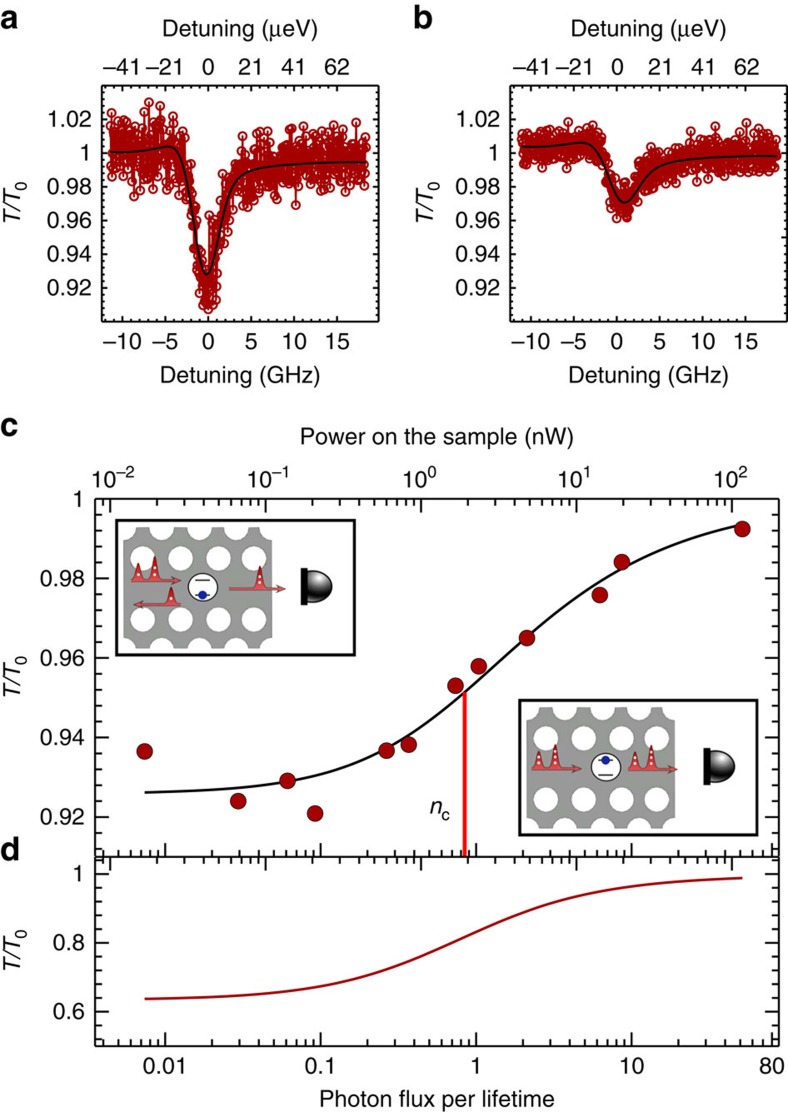
Non-linear response of a single quantum dot in a photonic-crystal waveguide. Examples of transmission spectra recorded at two different powers of (**a**) *P*=0.18 nW and (**b**) *P*=2.2 nW. (**c**) Transmission on resonance with the quantum dot versus incident photon flux relative to the emitter lifetime. The top axis shows the corresponding optical power applied to the sample. The solid line is a fit to the experimental data. The critical power that characterizes the saturation curve is indicated on the axis. The insets show the measurement geometry and illustrate that for weak excitation the quantum dot preferentially reflects while it becomes transparent at stronger excitation where two- and higher-photon components of the coherent state dominate. (**d**) Same as (**c**) but after deconvolution of the spectral diffusion and blinking.

The non-linear response of the quantum dot is investigated by recording the transmission as a function of excitation power. Two examples of transmission spectra are displayed in Fig. 2a,b for weak and intermediate powers, respectively. In this data set a transmission dip of ∼8% was recorded, limited by spectral diffusion, which was found to vary when the sample was heated up and subsequently cooled down. Figure 2c shows the transmission as a function of power inside the waveguide and displays a characteristic non-linear saturation behaviour. The data are modelled very well by the theory of ref. 30 (*cf*. Supplementary Notes 5–7 and Supplementary Figs 5 and 6 for a detailed account) for a coupling efficiency of *β*=85%, broadening by spectral diffusion of *σ*/*Γ*=3.6, blinking probability of *α*=0.43, and a pure dephasing rate describing the broadening of the zero-phonon line of *γ*_0_/*Γ*=0.79. Here the emitter decay rate *Γ*=2.5 ns^−1^ is obtained in time-resolved measurements leading to an independent measurement of the coupling efficiency of *β*∼96% (ref. 18). The two values are consistent since the *β*-factor extracted from resonant scattering experiments is effectively reduced compared with the value obtained from the dynamics by the presence of weak phonon sidebands due to incoherent Raman scattering processes^31^. The incoherently scattered photons are not distinguishable from the coherently scattered photons in time-resolved measurements, while they contribute differently in the present transmission experiment. To maximize the contrast of the signal, the intensity of the repump laser is varied between 250 pW and 1.2 nW for these measurements, see Supplementary Note 8 and Supplementary Fig. 7. Figure 2d shows the non-linear response after deconvolution of the spectral diffusion and blinking, which can be suppressed by implementing electrical gates. We extract a critical photon flux per lifetime of *n*_c_=0.81 characterizing the non-linear saturation curve, demonstrating that the non-linearity operates at the ultimate level of single photons. This corresponds to a characteristic switching energy of only ∼0.17 attojoule. For comparison, the state-of-the-art switching power in the classical optics is around several femtojoules per switching event^32^,^33^ and the observed non-linearity has a big potential for very low-power optical switching. The input power applied to the sample at the critical power is 1.9 nW, which implies that 23% of the excitation beam is successfully coupled to the quantum dot in the waveguide. Photonic waveguide non-linearities are particularly promising for obtaining non-linearities at low photon numbers; a detailed comparison to the case of cavity polaritons is presented in Supplementary Note 9 and Supplementary Fig. 8.

### Quantum nature of the non-linearity

**Figure 3:**
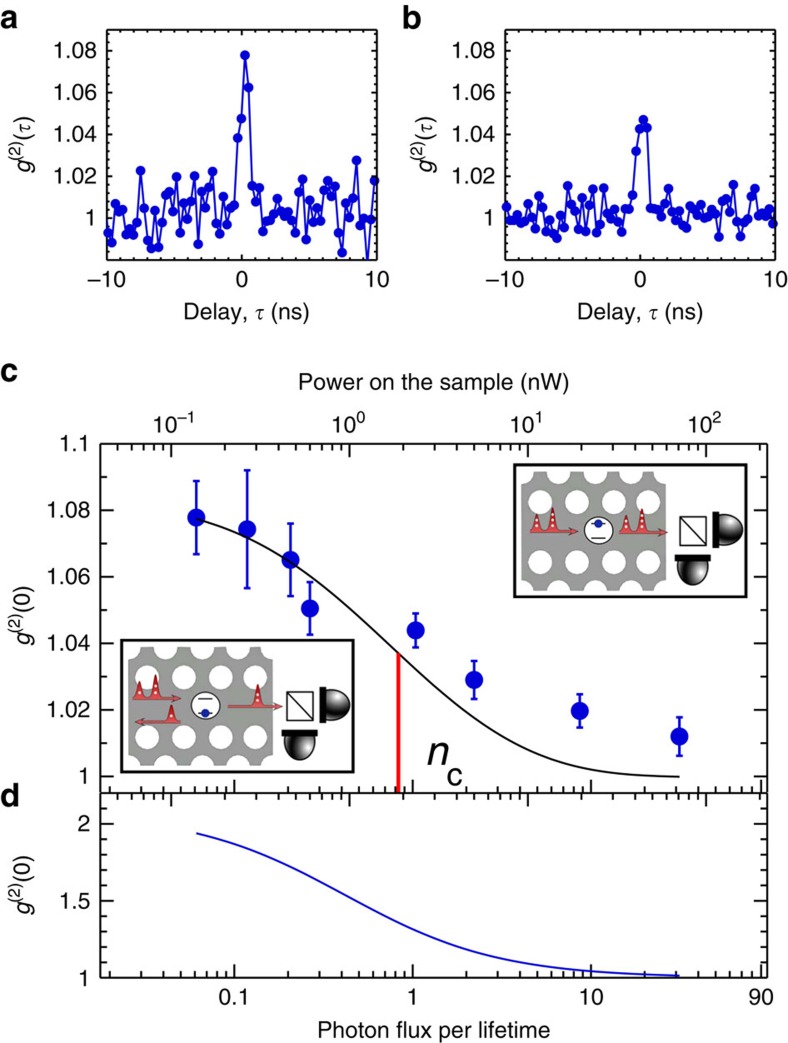
Photon statistics induced by the single-photon non-linearity. (**a**,**b**) Measurements of the autocorrelation function of the transmitted light recorded on resonance with the quantum dot for the conditions corresponding to Fig. 2a,b, respectively. (**c**) Power dependence of the autocorrelation function peak at 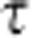
=0. The solid black line is a fit to the data. A maximum bunching of 8% is observed, which corresponds to 18% when accounting for the finite response time of the detection. The vertical line indicates the critical power. (**d**) Same as (**c**) but after deconvolution of the spectral diffusion and blinking.

By monitoring the photon statistics of the transmitted light, the quantum character of the non-linear response and the ability to generate photon–photon bound states are revealed. In the ideal case of *β*→1 and for a weak coherent state, the single-photon component is fully reflected while two- and higher-photon components are preferentially transmitted leading to photon bunching in the transmission^1^,^9^. Figure 3a,b show the experimental signature of photon bunching in the intensity autocorrelation function *g*^2^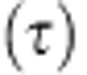 of the transmission as a function of the delay 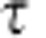. The peak centred at 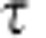
=0 is the experimental signature that two or more photons impinging on the quantum dot within its radiative lifetime interact leading to photon–photon bound states. We estimate a photon–photon bound-state contribution to the transmission probability of the two-photon component of ∼70%, which is the entangled part of the scattered light (see Supplementary Note 10 and Supplementary Fig. 9 for further details). The observed photon bunching is highly sensitive to decoherence processes^5^ and the experimental observation of this effect testifies that resonant photon scattering using quantum dots is highly coherent^34^. The amount of photon bunching is found to decrease with increasing power (*cf.* Fig. 3c) since two- and higher-photon components increasingly dominate the input state. At high excitation power, we observe *g*^2^(0)≈1, corresponding to the Poissonian statistics of a coherent state, that is, the recorded light is unaffected by the saturated quantum dot. This experiment demonstrates the basic operational principle behind photon sorting with applications in photonic quantum-information processing^8^. Several control experiments were conducted to confirm that the observed photon bunching is mediated by the quantum dot. For example, bunching of the photons vanishes when the laser is detuned far from the resonance of the quantum dot. We also verified that *g*^2^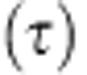 is a flat line when the repump laser is turned off, see Supplementary Note 11 and Supplementary Fig. 10 for more details. The observed degree of bunching is explained by three factors: pure dephasing and spectral diffusion of the quantum dot transition, as well as the limited instrument response function of the setup. After deconvolution of the latter, we obtain *g*^(2)^(0)=1.18. We emphasize that the two independent set of experimental data of Figs 2c and 3c are well explained by theory for the same set of parameters. The inherent potential of the system is illustrated in Fig. 3d, which shows the expected amount of photon bunching after deconvoluting the slow decoherence processes found in the experiment. For the parameters of the present experiment and in the weak excitation limit, we predict *g*^(2)^(0)∼2.1, which is mainly limited by the pure dephasing rate *γ*_0_. Importantly, such decoherence has been shown to give a minor contribution in single-photon indistinguishability measurements on quantum dots controlled by electrical gates^34^, that is, even more dramatic photon–photon scattering processes should be obtainable.

## Discussions

Having access to single-photon non-linearities may open new perspectives for processing both classical and quantum-information encoded in photons. In the classical regime, it enables constructing ultimately energy-efficient optical switches that are triggered by just a few quanta of light, which is required to outperform electronic transistors^33^. In the quantum regime, it may enable new and resource-efficient functionalities required for deterministic quantum-information processing with photons^35^. With spectral and spatial control of the quantum dots^36^ the present system can be scaled to obtain multiple quantum dots deterministically coupled to a photonic-crystal waveguide, each inducing a giant non-linearity. Such a complex quantum system may be exploited for advanced quantum simulations and to engineer novel quantum states of coupled light and matter^37^.

## Additional information

**How to cite this article:** Javadi, A. *et al*. Single-photon non-linear optics with a quantum dot in a waveguide. *Nat. Commun.* 6:8655 doi: 10.1038/ncomms9655 (2015).

## Supplementary Material

Supplementary Information Supplementary Figures 1-10, Supplementary Notes 1-11 and Supplementary References
