# Supplementary material for: Single-photon non-linear optics with a quantum dot in a waveguide
Source: Nat Commun. 2015 Oct 23;6:8655. doi: 10.1038/ncomms9655 (PMC4639909; doi:10.1038/ncomms9655)
Supplement: Supplementary Information — Supplementary Figures 1-10, Supplementary Notes 1-11 and Supplementary References [file ncomms9655-s1.pdf]

SUPPLEMENTARY INFORMATION

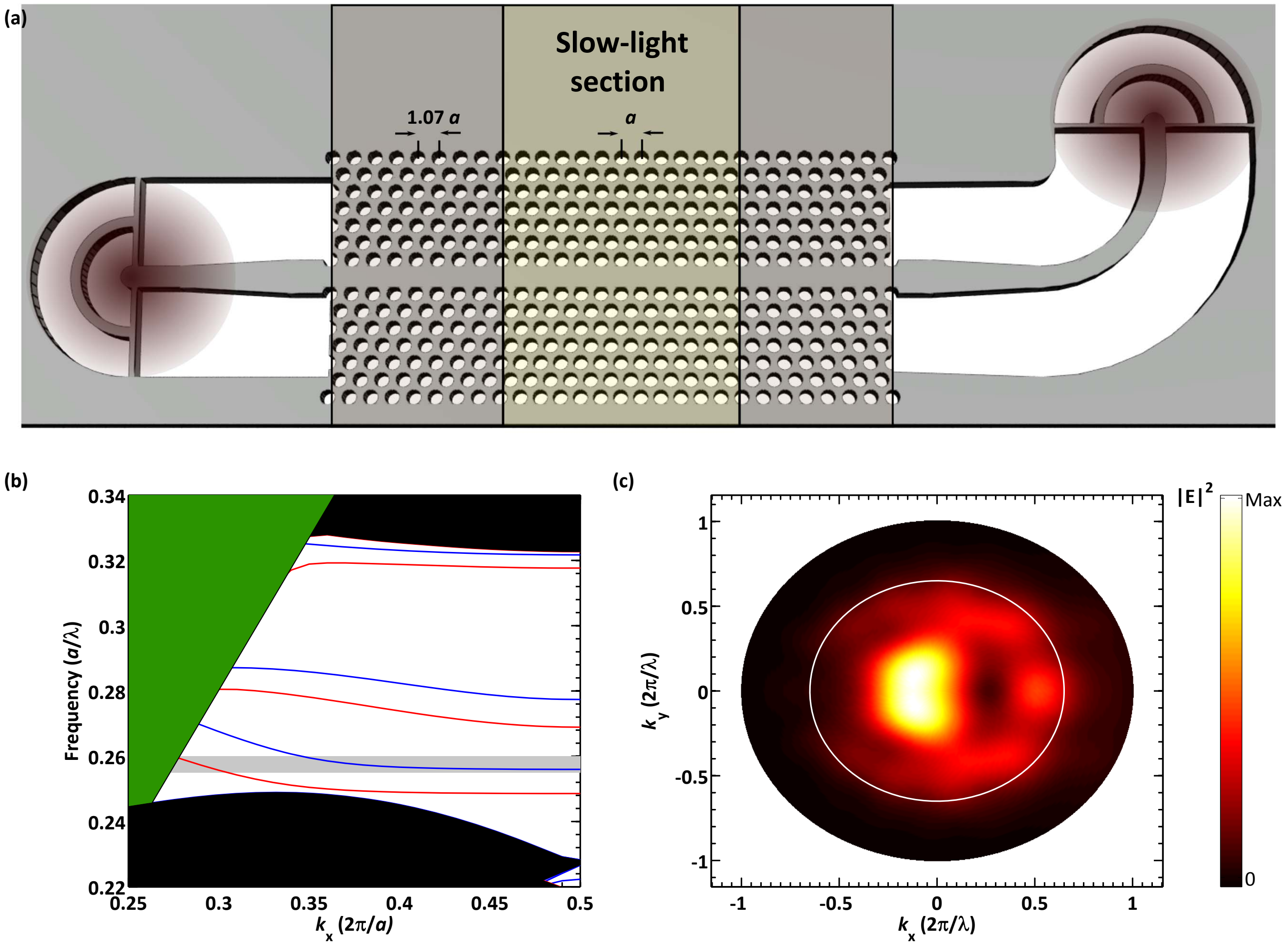

Supplementary Figure 1. **Detailed description of the sample.** (a) A sketch (not to scale) of the sample with the slow-light section labeled in the center of the sketch. (b) Bandstructure of the sample. The blue lines are the bands belonging to the slow-light section and the red ones belong to the weakly-dispersive section. The frequency range of interest for the transmission experiments is where the bands of the two waveguide regions overlap, which is shaded in gray. Finally, the green and black shaded regions mark the continuum of the radiation modes and the modes guided in the photonic-crystal slab, respectively. (c) The far-field pattern of the out-coupling gratings. The white circle indicates the circumference of the portion of the far-field pattern that is collected by an objective of NA = 0.65.

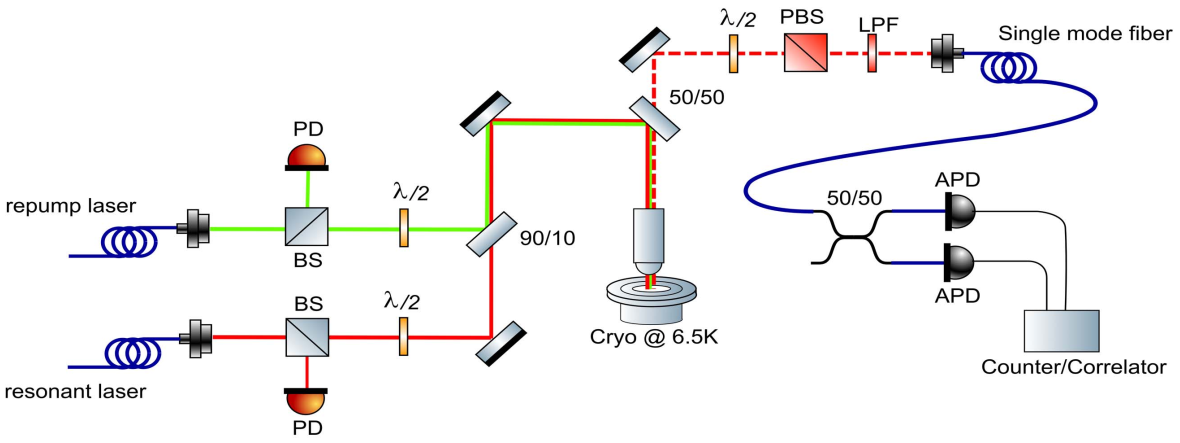

Supplementary Figure 2. **Simplified sketch of the optical setup.** The description of the setup is presented in the text.

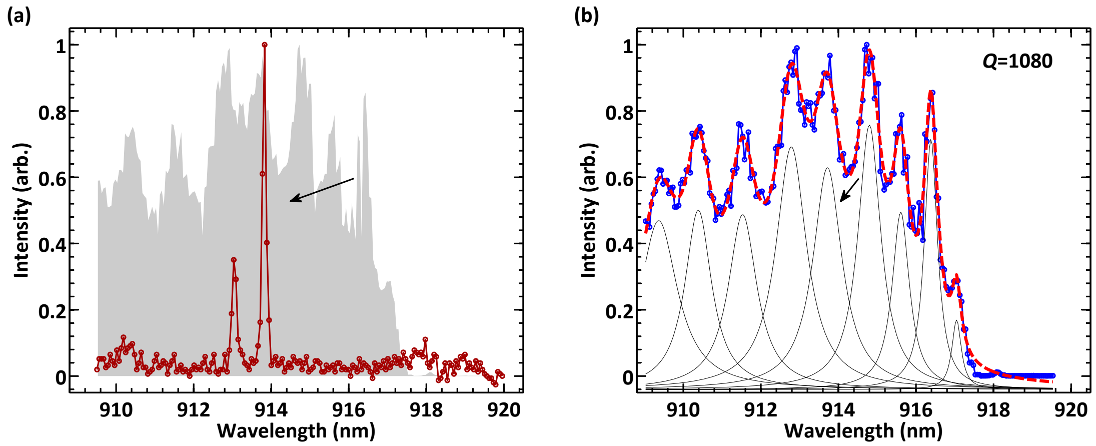

Supplementary Figure 3. **Emission and transmission spectrum of the waveguide.** (a) Emission spectrum of the sample under weak wetting layer excitation, solid red line and transmission spectrum of the waveguide under high resonant laser power, gray shaded region. (b) From lorentzian fits to the transmission spectrum the quality factor of the cavity is extracted to be 1080. At the final stage of the measurement the QD is close to resonance of the cavity.

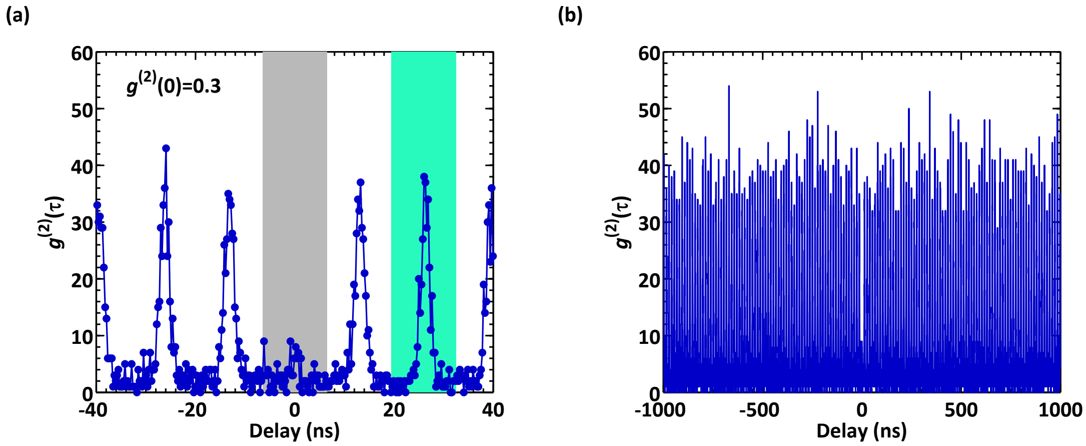

Supplementary Figure 4. **Autocorrelation measurements on the photons emitted from the QD.** (a) HBT correlation measurement on the exciton line under pulsed wetting layer excitation and operated close to the saturation power of the QD. The highly suppressed coincidence count at $\tau = 0$ proves that predominantly light from a single exciton is recorded. (b) Plot of the autocorrelation data on longer time scales. No signature of blinking is found under wetting layer excitation on the timescales of microseconds.

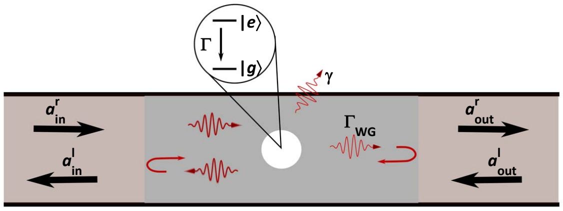

Supplementary Figure 5. **Schematic drawing of a QD in a 1D waveguide.** The partial reflection at the two ends of the waveguide are due to imperfect impedance matching between different interfaces inside the sample.

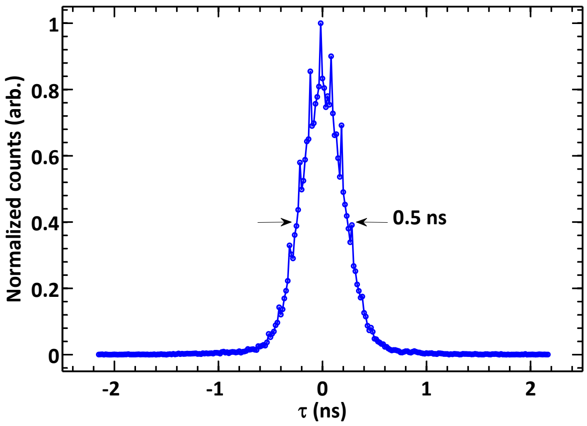

Supplementary Figure 6. **The instrument response function of the setup for $g^{(2)}$ measurements.** The instrument response function of the setup used to measure $g^{(2)}(\tau)$. The curve is obtained by sending 5 ps pulses with a wavelength of 920 nm through the measurement setup and recording the autocorrelation function. The full-width-at-half-maximum of the IRF is approximately 0.5 ns.

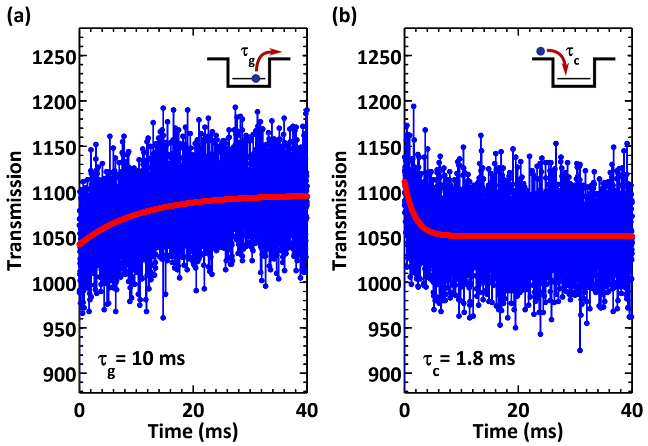

Supplementary Figure 7. **Dynamics of the ground state.** (a) Decay of the ground state to dark state as a function of time. At t=0 the repump laser is turned off and the change in transmission versus time is recorded. (b) Revival of the ground state from the dark state as a function of time. At $t = 0$ the repump laser is turned on and the change in transmission versus time is recorded. The solid red lines are single exponential fits to the data. We extract a ground state lifetime of 10 ms and a revival time constant of 1.8 ms.

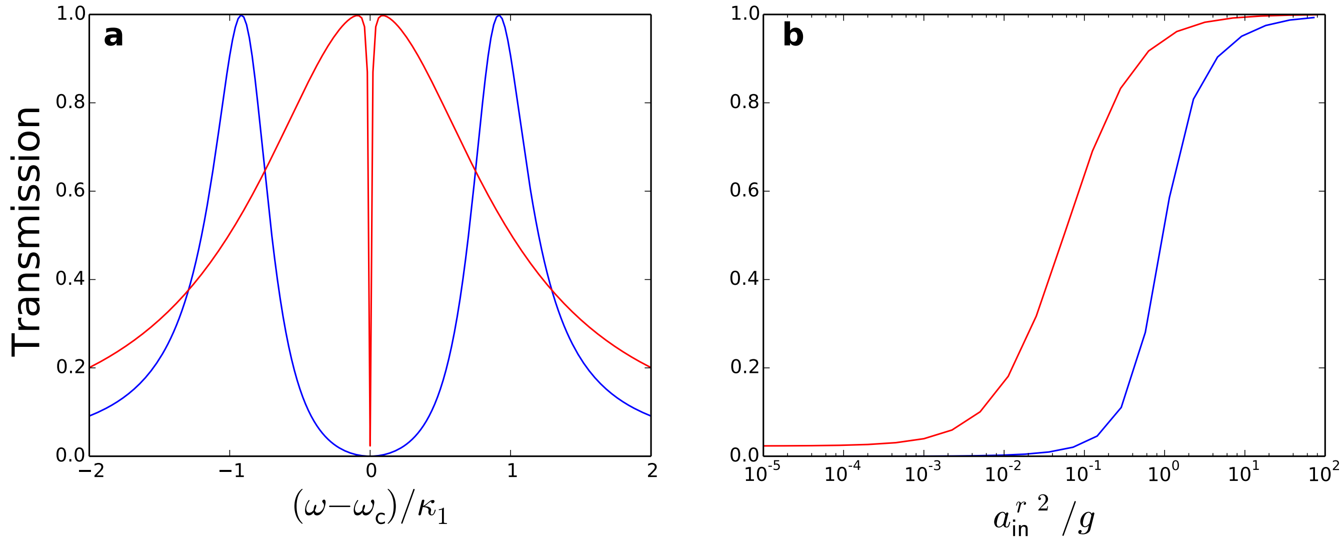

Supplementary Figure 8. **Power dependence of a strongly coupled QD-cavity system versus waveguide system.** Comparison of the transmission spectrum and input-output curve for a strongly coupled system (blue curves) and our system of a QD in a PCW (red curves). (a) Transmission as a function of detuning (normalized to the cavity width of the strongly coupled system). (b) Transmission versus number of photons per excited-state lifetime corresponding to ${a_{\rm in}^{\rm r}}^2/\Gamma$ for the PCW system and $2{a_{\rm in}^{\rm r}}^2/(\kappa+\gamma)$ for the strongly coupled system.

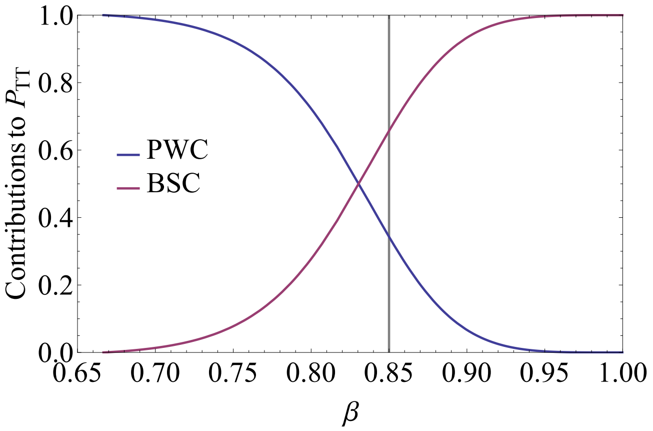

Supplementary Figure 9. **Plane-wave contribution versus bound-state contribution.** Comparison of the plane-wave and bound-state contributions normalized to the total transmission probability as a function of the β-factor. The bound state strongly depends on the relative spectral widths of the incoming photon state and the QD transition. From decay rate measurements and the specifications of our laser we can estimate a ratio of ~ 400 between their linewidths. The vertical line indicates $\beta = 85\%$, corresponding to the value extracted from the experiment.

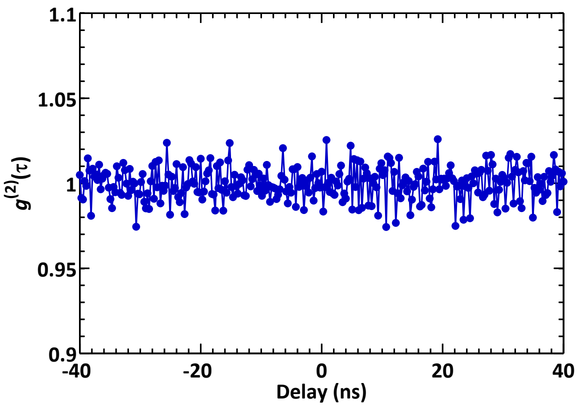

Supplementary Figure 10. **Autocorrelation function of the transmitted photons far from the resonance of the QD.** Statistics of the transmitted photons when the laser is detuned ∼20 GHz from the QD resonance. The excitation power is ∼0.27 nW. The flat $g^{(2)}(\tau)$ is a characteristic of a coherent state.

Supplementary Note 1. Detailed presentation of sample structure

In this section we outline the design parameters of the sample used for this experiment. The designed sample is shown schematically in Supplementary Fig. 1(a). It is composed of a slow-light photonic-crystal waveguide (PCW) section, which is coupled on both sides to weakly dispersive PCW sections. The slow-light sections feature a low group velocity in order to increase light-matter interaction while the weak-dispersion sections enable more efficient outcoupling from the structure. Each end of the PCW structure is coupled to regular bridge waveguides and outcoupling gratings. The membrane has a thickness of 160 nm and is composed of GaAs, which has a refractive index of $n = 3.464$ at 10 K. A layer of self-assembled InAs quantum dots (QD) are grown in the center of the membrane. Due to the nature of the self-assembly process, the QDs are located at random positions inside the sample. The increased coupling efficiency to the PCW relies on the strong suppression of coupling radiation modes together with an enhanced coupling into the waveguide mode, which makes it very robust to spatial and spectral dispersity meaning that most QDs are efficiently coupled. The photonic crystal consists of a triangular array of holes with radius 72 nm and lattice period $a = 235$ nm. A line defect is obtained by leaving out a row of holes, which forms the PCW. The two different PCW sections have the same structural parameters, with the exception that the lattice period is stretched from a to $1.07a$ in transiting from the slow-light section to the weakly-dispersive section. The frequency range of operation for the two waveguide sections is indicated in Supplementary Fig. 1(b). The slow-light section is 8.00 µm long, while each of the weakly-dispersive PCW sections is 15.6 µm long. A short slow-light section is used to minimize the effects of disorder-induced backscattering and out-of-plane scattering, as both of these increase with decreasing group velocity [1]. Even though the transition from the slow-light section of the PCW to the weakly-dispersive PCW is abrupt, it has been shown that evanescent modes mediate efficient coupling between two such waveguides [2]. The PCW is furthermore coupled to bridge waveguides with a width of $\sqrt{3}a$, corresponding to the center-to-center distance of the rows of holes adjacent to the waveguide. The bridge waveguides are tapered down to a width of λ/n over a length of $5 \times 1.07a$. The waveguide on the right in Supplementary Fig. 1(a) arcs through 90 degrees before it reaches the grating. The bend has a large radius of curvature of $5a$, which minimizes losses. The out-coupling gratings are based on the design presented in Ref. [3]. The gratings act as a second order Bragg reflector that couples light in the waveguide vertically to radiation modes. The grating is composed of alternating layers of concentric circles separated by $\lambda/2n$, where n is the refractive index that alternates between that of air and GaAs, and the design wavelength is $\lambda = 922$ nm. The design in Ref. [3] is improved by coupling into each grating from a bridge waveguide of width λ/n [4], which enhances the light intensity that falls in to the numerical aperture of the collection lens. The farfield radiation pattern is illustrated in Supplementary Fig. 1(c). The white circle indicates the numerical aperture of the collection lens (NA = 0.65). Numerical simulations show that $\sim 90\%$ of the light is collected by the first lens. The two gratings are arranged to have orthogonal polarization in the far-field, which results in the light scattered at the in-coupling grating and the light collected from the out-coupling grating having orthogonal polarization and can therefore be easily separated by the use of polarizers.

Supplementary Note 2. Experimental setup and procedure

Supplementary Fig. 2 shows the experimental setup. Two continuous wave lasers are used throughout the experiments presented in the manuscript. These lasers are focused onto the sample through a single microscope objective (NA = 0.65). One laser is tuned to 851 nm, which is near the edge of the wetting layer, labeled *repump laser* in Supplementary Fig. 2. This laser is focused around the center of the slow light region, as indicated by the green shaded region in Fig. 1b of the manuscript. The purpose of the repump laser is to revive the QD transition. The physical interpretation of the effect of the repump laser is given in Sec. Supplementary Note 8. The fiber labeled *resonant laser* couples a narrow band laser, (~ 1 MHz). This laser is coupled into and out of the waveguide using the grating couplers, see Supplementary Fig. 1(a). Both of the lasers exit the fibers with a linear polarization. The polarization axis can be rotated to align with any desired direction by using the half-wave plates ($\lambda/2$) positioned before the cryostat.

For the transmission measurements shown in Fig. 2 of the main manuscript, the resonant laser spectrally sweeps through the resonance of the QD transition and the transmission through the structure is recorded as a function of laser wavelength. The transmitted light is coupled into a single-mode fiber, which serves as a spatial filter only collecting the light scattered out through the out-coupling grating. Further extinction of excitation laser scattered at the in-coupling grating is achieved through the combination of the half-wave plate ($\lambda/2$) and the polarizing beam splitter (PBS) in the collection arm. These two components serve as a high quality polarization filter, which is aligned to the far-field polarization of the out-coupling grating. Finally, a long-pass filter (LPF) is used to suppress any residual scattering from the repump laser. While scanning the resonant laser, the repump laser is modulated

between on and off at a frequency of 2.5 Hz using an acusto-optical modulator. The counts on the avalanche photodiode (APD) are integrated during the bright and dark periods of the repump laser. The transmission spectrum is constructed using the ratio between the counts in the bright period and the counts in the dark period (T/T_0).

The above described measurements have focused on the transmitted photon flux, but additional information can be gained from measurements of the second-order correlation function, $g^{(2)}(\tau)$, of the transmitted light. For these measurements the single-mode fiber used in collection is connected to a balanced fiber-beam-splitter, which has each output port connected to an APD. The detection events on each APD are recorded and given a time tag, which is used to generate the experimental autocorrelation function, presented in Fig. 3 of the manuscript. During the entire measurement the resonant laser is fixed at the central position of the transmission dip and the repump laser remains in the on state. The power of the resonant laser at the fiber output is stabilized during the experiments using the voltages of the photodiode (PD) as a feedback signal.

Both types of measurements discussed above are performed for a large range of incident resonant powers and the saturation behavior is shown in Fig. 2(c) and Fig. 3(c) of the main manuscript. During these experiments the power is measured at the PD and by characterizing the losses of all the optical components this is used to find the power incident on the sample. In order to maximize the lifetime of the ground state, the intensity of the repump laser is varied between 250 pW to 1.2 nW. This is more than 2 orders of magnitude below the saturation power we observe under CW excitation. In these power regimes, the repump laser does not lead to significant emission.

Supplementary Note 3. Characterizing the nanostructure and finding a suitable transition

The transmission of the nanostructure is found from a sweep of the resonant laser. Performing this sweep at high power ensures that only the nanostructure is probed, and the spectral dependence of the normalized transmission is indicated by the gray shaded region in Supplementary Fig. 3(a). From the clearly visible cutoff in the transmission the bandedge of the slow-light region is found to be at 917 nm. The emission spectrum of the sample for low power wetting layer excitation recorded on the slow-light region of the PCW is shown as the solid red curve Supplementary Fig. 3(a). The exciton chosen for resonant spectroscopy is marked by the black arrow. Supplementary Fig. 3(b) shows the Lorentzian fits to the Fabry–Perot resonances in the transmission spectrum. The transmission peak that is closest to our target exciton is marked by a black arrow. From the multiple Lorentzian fits to the spectrum we extract the quality factor (Q) of each resonance. The Q-factor for the resonance closest to our target QD is found to be 1080.

Time-resolved emission experiments are furthermore carried out using pulsed wetting-layer excitation in order to determine the coupling efficient. The resulting emission curves are best modelled by a single exponential decay with $\Gamma = 2.5\,\text{ns}^{-1}$. This indicates that the transition is likely a charged-exciton since the two orthogonal linear dipoles of the neutral exciton typically exhibit asymmetric decay rates in PCWs [5]. In addition, neutral excitons can be in an optically dark state [6], which leads to bi-exponential decay curves. Following the procedure described in [5], the β-factor for the transition is extracted to be 96%.

Supplementary Note 4. Confirming the single-photon nature of the emission

To ensure that the exciton line originates from a single QD, we carried out correlation measurements using a Hanbury-Brown and Twiss (HBT) setup under wetting layer excitation. We excited the QD around its saturation power, $\sim 50\,\text{nW}$. The collected light was sent through a monochromator to filter out the emission from other excitonic lines in the spectrum. The area around zero delay, indicated by the gray shaded region in Supplementary Fig. 4(a), is compared to the area of pulses far from zero delay, e.g., the green shaded region. Taking the ratio between these two areas gives a conservative estimate of $g^{(2)}(0) = 0.3$. Plotting the same autocorrelation data over a timescale of 2 µs, we observe no clear indications of any emitter blinking processes.

Supplementary Note 5. Theoretical analysis

In this section we analyse the dynamics of the QD in the PCW. Supplementary Fig. 5 illustrates the physical picture of our sample: a two level system is placed in a coupled cavity-waveguide system. The effect of the cavity arises due to the residual reflections at the interfaces in the composite waveguide. We follow the theory in [7]. The time evolution of the atomic operators are

$$\dot{s}_- = i\Delta\omega s_- - \frac{\Gamma_{\mathrm{WG}}}{2}\left(\frac{1}{f} + t_0\right) s_- - 2i\Omega t_0 s_{\mathrm{z}},$$
$$\dot{s}_{\mathrm{z}} = -\Gamma_{\mathrm{WG}}\left(\mathrm{Re}\,(t_0) + \frac{\gamma}{\Gamma_{\mathrm{WG}}}\right)(s_{\mathrm{z}} + 1) + i\left(\Omega t_0 s_-^* - c.c.\right), \tag{1}$$

with

$$f = \frac{\Gamma_{\mathrm{WG}}}{2\gamma_0 + \gamma},$$
$$t_0 = \frac{1}{1 + \frac{i(\delta - \Delta\omega)}{\kappa}}. \tag{2}$$

Here s_- and s_{z} are the expectation values of the atomic operators $\hat{s}_- = |g\rangle\langle e|$ and $\hat{s}_{\mathrm{z}} = (|e\rangle\langle e| - |g\rangle\langle g|)/2$, respectively. The decay rate of the emitter to the waveguide mode and radiation modes are given by Γ_{WG} and γ respectively, γ_0 is the pure dephasing rate of the emitter, δ is the detuning between Fabry–Perot resonance of the waveguide and the QD, $\Delta\omega$ is the detuning between the QD resonance and the resonant laser ($\Delta\omega = \omega_{\mathrm{laser}} - \omega_{\mathrm{QD}}$), κ is the coupling rate of the Fabry–Perot resonance to the waveguide mode, and Ω is the Rabi frequency of the resonant field.

The amplitudes of the electric fields on the output port (right side in Supplementary Fig. 5) can be expressed in terms of the amplitudes of the electric fields in the input port (left side in Supplementary Fig. 5) as:

$$a_{\mathrm{out}}^{\mathrm{r}} = -t_0 a_{\mathrm{in}}^{\mathrm{r}} - i\sqrt{\frac{\Gamma_{\mathrm{WG}}}{2}} t_0 s_-,$$
$$a_{\mathrm{in}}^{\mathrm{l}} = (1 - t_0)\, a_{\mathrm{in}}^{\mathrm{r}} - i\sqrt{\frac{\Gamma_{\mathrm{WG}}}{2}} t_0 s_-, \tag{3}$$

where we have set $a_{\mathrm{out}}^{\mathrm{l}} = 0$. The input field is related to the driving Rabi frequency through $\Omega = a_{\mathrm{in}}\sqrt{\Gamma_{\mathrm{WG}}/2}$. The steady-state solution of the equations of motion can be readily derived [7]:

$$s_- = -\frac{4}{\Gamma_{\mathrm{WG}}} \frac{i\Omega t_0 s_{\mathrm{z}}}{t_0 + \frac{\gamma}{\Gamma_{\mathrm{WG}}} + \frac{2\gamma_0}{\Gamma_{\mathrm{WG}}} - \frac{2i\Delta\omega}{\Gamma_{\mathrm{WG}}}},$$
$$s_{\mathrm{z}} = -\frac{1}{2}\frac{1}{1 + \Omega^2/\Omega_{\mathrm{c}}^2}. \tag{4}$$

and

$$\Omega_{\mathrm{c}}^2 = \frac{|-2i\Delta\omega - (\gamma + t_0\Gamma + 2\gamma_0)|^2\,[\gamma + \Gamma\,\mathrm{Re}(t_0)]}{8|t_0|^2[\gamma + 2\gamma_0 + \Gamma\,\mathrm{Re}(t_0)]}, \tag{5}$$

where Ω_{c} corresponds to the excitation power where $s_{\mathrm{z}} = -\frac{1}{4}$ and $\Gamma = \Gamma_{\mathrm{WG}} + \gamma$. For the purpose of evaluating the nonlinearity in terms of the number of input photons, it is instructive to define a parameter $n_\tau = 2\frac{\Omega^2}{\Gamma^2}$. This parameter corresponds to the mean photon flux in the input field per lifetime of the emitter. The critical photon number can then be defined as $n_{\mathrm{c}} = 2(\frac{\Omega_{\mathrm{c}}}{\Gamma})^2$. For a QD in resonance with the cavity and $\Delta\omega = 0$, the critical photon number is:

$$n_{\mathrm{c}} = \frac{1 + 2\beta\gamma_0/\Gamma}{4\beta^2}. \tag{6}$$

where $\beta = \frac{\Gamma_{\mathrm{WG}}}{\gamma + \Gamma_{\mathrm{WG}}}$ is the collection efficiency of the photons scattered from the QD. The critical excitation power P_{c} corresponds to $\hbar\omega n_{\mathrm{c}}\Gamma$. The transmission and reflection coefficients of the field can be extracted as:

$$t = \frac{\langle a_{\mathrm{out}}^{\mathrm{r}}\rangle}{\langle a_{\mathrm{in}}^{\mathrm{r}}\rangle},$$
$$r = \frac{\langle a_{\mathrm{in}}^{\mathrm{l}}\rangle}{\langle a_{\mathrm{in}}^{\mathrm{r}}\rangle}. \tag{7}$$

The transmission coefficient of the system for $\Delta\omega = 0$ and $\delta = 0$ can be simplified to:

$$t = 1 - \frac{\Gamma\beta}{(\Gamma + 2\gamma_0\beta)(1 + n_\tau/n_{\mathrm{c}})}. \tag{8}$$

It is important to note that Eqs. (7) are only valid for the coherent part of the transmitted field, which applies for weak excitation. For higher excitation powers the transmitted field is a sum of coherent and incoherent components. The incoherent part of the scattering from the QD can be extracted as:

$$\frac{P_{\text{incoh}}}{{a_{\text{in}}^{\text{r}}}^2} = 1 - \left(|t|^2 + |r|^2 + \gamma|\langle s_-\rangle|^2/{a_{\text{in}}^{\text{r}}}^2\right), \tag{9}$$

where a_{in}^{r} is chosen to be a real function. The total transmission is:

$$T = |t|^2 + \beta P_{\text{incoh}}/2{a_{\text{in}}^{\text{r}}}^2. \tag{10}$$

Finally the transmission spectrum of the system can be obtained from:

$$\frac{T}{T_0} = \frac{T}{|t_0|^2}. \tag{11}$$

The autocorrelation functions for the transmitted electric field can be related to those of the atomic operators as:

$$G^{(2)}(\tau) = \langle a_{\text{out}}^{\text{r}\dagger}(0)a_{\text{out}}^{\text{r}\dagger}(\tau)a_{\text{out}}^{\text{r}}(\tau)a_{\text{out}}^{\text{r}}(0)\rangle. \tag{12}$$

Since the emitter is close to the center of the resonance of the cavity we neglect the effect of the cavity in calculation of $G^{(2)}(\tau)$ and substitute $|t_0| = 1$. $G^{(2)}(\tau)$ can then be written as:

$$\begin{aligned} G^{(2)}(\tau) =& {a_{\text{in}}^{\text{r}}}^4 - 4\sqrt{\frac{\Gamma_{\text{WG}}}{2}}{a_{\text{in}}^{\text{r}}}^3\,\text{Im}\left[\langle s_-(0)\rangle\right] + \Gamma_{\text{WG}}{a_{\text{in}}^{\text{r}}}^2\left(\frac{1}{2} + \langle s_{\text{z}}(0)\rangle\right) + \\ & \Gamma_{\text{WG}}{a_{\text{in}}^{\text{r}}}^2\,\text{Re}\left[\langle {s_-}^\dagger(0)s_-(\tau) - {s_-}^\dagger(0)s_-(\tau)^\dagger\rangle\right] + \Gamma_{\text{WG}}\sqrt{\frac{\Gamma_{\text{WG}}}{2}}a_{\text{in}}^{\text{r}}\,\text{Im}\left[\langle {s_-}^\dagger(0)s_{\text{z}}(\tau)\rangle\right] \\ & - \frac{\Gamma_{\text{WG}}}{2}\sqrt{\frac{\Gamma_{\text{WG}}}{2}}a_{\text{in}}^{\text{r}}\,\text{Im}\left[\langle s_-(0)\rangle\right] + \Gamma_{\text{WG}}\sqrt{\frac{\Gamma_{\text{WG}}}{2}}a_{\text{in}}^{\text{r}}\,\text{Im}\left[\langle {s_-}^\dagger(0)s_-^\dagger(\tau)s_-(0)\rangle\right] + \\ & \left(\frac{\Gamma_{\text{WG}}}{2}\right)^2\langle {s_-}^\dagger(0)s_{\text{z}}(\tau)^\dagger s_-(0)\rangle + \frac{1}{2}\left(\frac{\Gamma_{\text{WG}}}{2}\right)^2\left(\frac{1}{2} + s_{\text{z}}(0)\right), \end{aligned} \tag{13}$$

and the normalized auto correlation function can be written as:

$$g^{(2)}(\tau) = \frac{G^{(2)}(\tau)}{\left\langle\left(a_{\text{in}}^{\text{r}} - i\sqrt{\frac{\Gamma_{\text{WG}}}{2}}{s_-}^*(\tau)\right)\left(a_{\text{in}}^{\text{r}} + i\sqrt{\frac{\Gamma_{\text{WG}}}{2}}s_-(\tau)\right)\right\rangle^2}. \tag{14}$$

It is possible to obtain an analytic solution of Eq. (14) for the case of $\tau = 0$. However, since the measurements are convoluted with the instrument response function (IRF) of the detector and broadened by spectral diffusion we need the time and detuning dependence of $g^{(2)}(\tau)$, in order to compare experimental results and theoretical predictions, even if we are just interested in the value of $g^{(2)}(0)$. We apply the quantum regression theorem to the Bloch equations and obtain the equations of motion for the two and three times operator products entering in Eq. (13). Subsequently, we numerically solve these equations and extract $g^{(2)}(\tau)$. The result is convoluted with a Gaussian function to account for the spectral diffusion of the QD transition to get $g^{2*}(\tau)$, see the next section for the details. Finally, we convolute the $g^{2*}(\tau)$ with the IRF of the detection, which is shown in Supplementary Fig. 6.

Supplementary Note 6. Effect of blinking and spectral diffusion

Charge and spin fluctuations in the environment of the QD lead to spectral diffusion and blinking of the QD transitions [8, 9] and have been reported by several groups [8, 10–12]. The time scale of these fluctuations is found to be long compared to coherence times of the QD transition, but short compared to our measurement time of the resonant transmission experiment. The spectral diffusion can be modeled by including a random detuning in the transition frequency of the QD, that is $\omega = \omega_0 + \delta_0$ where ω_0 is the resonance frequency of the QD and δ_0 is a random variable with a Gaussian probability distribution:

$$P(\delta_0) = \frac{1}{\sqrt{2\pi\sigma^2}}e^{-\delta_0^2/2\sigma^2}. \tag{15}$$

In order to obtain the time averaged effect of spectral diffusion on the transmitted light intensity and its statistics, Eqs. (11) and (14) are convoluted with Eq. (15):

$$T^{*} = \int_{-\infty}^{\infty} \mathrm{d}\delta_0 T(\Delta\omega - \delta_0) P(\delta_0), \tag{16}$$

$$g^{2*}(0) = \int_{-\infty}^{\infty} \mathrm{d}\delta_0 g^{(2)}(0, \Delta\omega - \delta_0) P(\delta_0). \tag{17}$$

Blinking of the QD can be characterized as the ratio between the time the QD spends in the dark state over the sum of the time spent in the bright and the dark states (α). The effect of blinking can be modeled as:

$$T_{\mathrm{exp}} = (1-\alpha)T^{*} + \alpha \times 1, \tag{18}$$

$$g^{(2)}_{\mathrm{exp}}(\tau) = (1-\alpha)g^{(2)*}(\tau) + \alpha \times 1. \tag{19}$$

Physically, these equations can be interpreted as follows: when the QD is in the dark state (with probability of α) the transmitted light is the coherent laser state. When the QD is in the bright state (with probability $(1-\alpha)$) the transmission spectrum and the autocorrelation function are given by equations (16) and (17)[13, 14]. Hence a weighted average of the two states gives the experimentally observed quantity.

Supplementary Note 7. Fitting the experimental data

In the present section it is explained in detail how the experimental data presented in the main manuscript are modelled using the theory introduced in the previous section. The experimental data in Figs. 2(a) and (b) are modeled with Eq. (18) and the resulting model curves are shown as solid black lines in the respective figures. The parameters governing the nonlinear response of the system are Γ, β, σ, α, γ_0, and δ. Additionally, an overall scaling factor, A_0, relates the power incident on the sample to the photon flux at the position of the QD. A_0 includes all loss processes relating to the in coupling of the resonant laser to the PCW and the subsequent propagation to the position of the QD. From high-power (cf. Supplementary Fig. 3) and low-power transmission spectra, we extract $\delta \approx 70\,\mathrm{GHz}$. The detuning is a free parameter for all transmission spectra, however it is found to vary within a limited range such that it has a negligible effect on the actual shape of the transmission curve.

The determination of the governing parameters in the model proceeds as follows. From independent time-resolved measurements, we extract a QD decay rate of $\Gamma = 2.5\,\mathrm{ns}^{-1}$. The scaling factor, A_0, is defined as $\langle n \rangle = A_0 P_{\mathrm{input}}$, where P_{input} is the power measured on the sample. Eq. (8) shows that the scaled transmission is independent of the power for excitation powers much below the critical power and therefore independent of the scaling factor. Consequently, the four low-power data points in Fig. 2(c) are modelled initially and we extract an average value of ($\beta = 0.85 \pm 0.18$) from this analysis.

While A_0 and β are independent of the power of the resonant field, spectral diffusion, pure dephasing, and blinking may vary. This is partly due to the fact that we optimized the power of the repump laser as the resonant laser power was increased. σ, γ_0, α, and A_0 are obtained from an iterative fitting routine composed of two main steps. In step one we fix α and recursively fit the data in Fig. 2(c) and Fig. 3(c) while varying the other free parameters. This is done in the following way: we start with a guess for γ_0 and fit the data in Fig. 2(c) to extract σ and A_0. We then fit the data in Fig. 3(c) with γ_0 as free, while using σ and A_0 extracted in the previous step. Next, we use the value of γ_0 from the last step and refit the data for σ and A_0. We repeat this process until a steady state for γ_0, σ, and A_0 is reached. This concludes step one. In step two we model the individual transmission curves at different powers by fixing A_0 and γ_0 and fitting α and σ for each transmission curve. The spectral diffusion is left as a free parameter in this round since it plays a dominant role for the width of the resonance. The average value of the spectral diffusion, $\sigma/\Gamma = 3.9 \pm 0.24$, extracted in this step agrees well with that extracted in the other step (see below). We find that α varies between 0.25 and 0.5 for different resonant powers. We take the average of α and feed it back into step one of the recursive fitting routines. From the complete recursive routine, we obtain $\gamma_0/\Gamma = 0.79 \pm 0.23$, $\sigma/\Gamma = 3.6 \pm 0.45$, $A_0 = 1.84 \pm 0.7 \times 10^{7}$, and $\alpha = 0.4 \pm 0.05$, where we note that these numbers are to be considered as average values over the investigated power range.

The final results of modelling the experimental data are shown as black curves in Fig. 2(c) and Fig. 3(c). The plot is Figs. 2(d) and 3(d) corresponds to the deconvoluted response where spectral diffusion and blinking have been neglected ($\sigma = \alpha = 0$). The model of the data of Fig. 1(d) corresponds to Eq. (16) for the parameters $\Gamma = 4.8\,\mathrm{ns}^{-1}$, $\beta = 0.88$, and $\sigma/\Gamma = 3$.

Supplementary Note 8. Measurement of the ground state lifetime and revival time

The presence of access charges and defect sites in the environment of the QD implies that the ground state may become meta-stable, since carriers may tunnel in and out of the QD [9, 13]. In order to activate this meta-stable state a repump laser is applied as described above. The resonant laser is tuned to be exactly on resonance and we extract the evolution of the ground state by modulating the repump laser at 2 Hz. The transmission of a weak resonant laser is recorded versus time in the bright and dark periods of the repump laser, see Supplementary Fig. 7. The recorded histograms are fitted with single exponential functions to extract the lifetime of the ground state, τ_g, and the time associated with capturing a charge in the QD, τ_c.

Supplementary Note 9. Comparison to strong-coupling nonlinearity

In this section we briefly compare the nonlinear response of the QD in a PCW to that of an emitter strongly coupled to a cavity, i.e., a 1D artificial atom to a cavity polariton. We compare the two systems by neglecting spectral diffusion and dephasing. State-of-the-art performance of a QD strongly coupled to a cavity corresponds to the parameters $(g_1, \kappa_1, \gamma_1)/2\pi = (22, 24, 0.01)$ GHz, as reported in Ref. [15]. For comparison, we use the parameters in our PCW system: $\beta = 0.85$, $\Gamma = 2.5\,\mathrm{ns}^{-1}$, and $Q = 1080$, from which we compute rates for the Jaynes-Cummings model. The Jaynes-Cummings Hamiltonian governing the system in both regimes rotating at the frame of the drive laser is

$$H/\hbar = (\delta - \Delta\omega)\hat{a}^\dagger\hat{a} - \Delta\omega\hat{s}_+\hat{s}_- + ig(\hat{a}^\dagger\hat{s}_- - a\hat{s}_+) - ia^{\mathrm{r}}_{\mathrm{in}}\sqrt{\frac{\kappa}{2}}(\hat{a}^\dagger - a). \tag{20}$$

Since we are considering a strongly coupled system we cannot use the theory in Ref. [7] and must solve for the dynamics numerically. We use freely available software [16] to solve for the steady-state correlation functions and spectral density of the transmitted field. From this we first compute the transmission in the low power regime as a function of detuning for the two systems. This is shown in Supplementary Fig. 8(a). We note that both systems display a spectrum exhibiting a dipole-induced reflection, where the depth of the central dip scales with the cooperativity of the photon-QD coupling. The width of the two systems scales differently: in the strongly coupled system the width is $2g$, while for the PCW the width is given by the decay rate Γ.

In Supplementary Fig. 8(b) we show the transmission for different driving strengths normalized by the excited-state lifetime of the systems $1/\Gamma$ and $(\kappa + \gamma)/2$ for the PCW and strongly-coupled systems, respectively. Both systems exhibit saturation behaviour with a contrast depending on the cooperativity/β-factor, however, the curves have different critical photon numbers and different shapes. For the PCW system, as shown in Eq. (6), the critical photon number (for $\gamma_0 = 0$) is $n_c = 1/4\beta^2$ and from Eq. (8) it can be shown that the slope of the saturation curve is fixed.

For the strongly coupled system both the slope of the curve and the critical photon number are different to the PCW system. From numerical calculations we have found that both the slope of the saturation curve and the critical photon number per lifetime scale with $g^2/(\kappa+\gamma)^2$. This difference in the saturation behaviour is related to the underlying physics of the two systems. In the PCW system an incident coherent state scatters off a single QD and, when the incident photon flux becomes greater than the decay rate, the two level system is saturated by the incident field. In the strongly-coupled system the incident coherent state interacts with a Jaynes-Cummings ladder of polariton states. The contrast of the splitting in the Jaynes-Cummings ladder is proportional to $g/(\kappa+\gamma)$. Higher levels in the Jaynes-Cummings ladder have a smaller splitting per photon, and thus as the power of the incident coherent state increases it couples to higher rungs of the ladder and is transmitted. Therefore, since the contrast of the polariton splitting is proportional to $g/(\kappa + \gamma)$, higher incident powers are required to saturate systems with stronger light-matter coupling. Since the PCW system has a lower critical photon number it may be more advantageous for narrow bandwidth applications requiring nonlinear saturation at low photon numbers.

Supplementary Note 10. Estimating the two-photon bound-state contribution

The observed bunching in the second order-coherence measurements, presented in Fig. 2 of the main manuscript, shows that the QD induces strong temporal correlations between the transmitted photons. The correlations occur on the time scale of the QD lifetime, which is a signature of the formation of photon-photon bound states.

In the following we demonstrate in a simplified model how the bound states lead to an enhanced transmission probability. For simplicity, we only consider the two-photon component of the incident field and ignore pure dephasing.

We calculate the total scattering matrix, S, which relates the incoming state to the outgoing state. We only consider the case of both photons being transmitted corresponding to

$$|2_{\mathrm{in}}\rangle \overset{S^{(\mathrm{tt})}}{\to} \left|2_{\mathrm{out}}^{(\mathrm{tt})}\right\rangle, \tag{21}$$

where $S^{(\mathrm{tt})}$ is the relevant part of the scattering matrix. The outgoing state can be split into the plane wave (PW) and bound-state (BS) parts

$$\left|2_{\mathrm{out}}^{(\mathrm{tt})}\right\rangle = \left|2_{\mathrm{PW}}^{(\mathrm{tt})}\right\rangle + \left|2_{\mathrm{BS}}^{(\mathrm{tt})}\right\rangle, \tag{22}$$

which correspond to the separable and non-separable contributions of the state [17–19]. The total transmission probability of an incoming two-photon state is given by

$$P_{\mathrm{tt}} = \left\langle 2_{\mathrm{out}}^{(\mathrm{tt})}\middle| 2_{\mathrm{out}}^{(\mathrm{tt})}\right\rangle = \left\langle 2_{\mathrm{PW}}^{(\mathrm{tt})}\middle| 2_{\mathrm{PW}}^{(\mathrm{tt})}\right\rangle + \left\langle 2_{\mathrm{PW}}^{(\mathrm{tt})}\middle| 2_{\mathrm{BS}}^{(\mathrm{tt})}\right\rangle + \left\langle 2_{\mathrm{BS}}^{(\mathrm{tt})}\middle| 2_{\mathrm{PW}}^{(\mathrm{tt})}\right\rangle + \left\langle 2_{\mathrm{BS}}^{(\mathrm{tt})}\middle| 2_{\mathrm{BS}}^{(\mathrm{tt})}\right\rangle, \tag{23}$$

where the first term is the plane-wave contribution and the remaining three terms are the bound-state contribution. The formation of the bound state strongly depends on the spectral width of the incoming state relative to the linewidth of the transition. In Supplementary Fig. 9 we consider a spectral width of ~ 1 MHz for the incoming state, corresponding to the linewidth of our laser. For the QD decay rate of $\Gamma = 2.5\,\mathrm{ns}^{-1}$ measured in the experiments this corresponds to a factor ~ 400 between the linewidths of the incident wave packet and the QD. At $\beta = 85\%$ we see that roughly $\sim 70\%$ of the total transmission probability of the two-photon state arises from the photon-photon bound-state contribution.

Supplementary Note 11. Cross checking the validity of the $g^{(2)}(\tau)$ measurements

We have carried out several measurements to ensure that the observed photon bunching in the transmission of the waveguide is due to interaction of the laser field with the QD rather than, e.g., thermal radiation, which could also lead to bunching. As a first check, we detuned the resonant laser from the transmission dip by $\sim$20 GHz and measured the statistics of the transmitted photons. Supplementary Fig. 10 shows the measured $g^{(2)}(\tau)$. As expected from a coherent field the $g^{(2)}(\tau)$ is unity for all τ. We repeated this type of measurement for several different powers with the same result. As a second check, we tuned the resonant laser to the transmission dip, turned off the repump laser, and repeated the $g^{(2)}(\tau)$ measurement. Note that when the repump laser is turned off, the QD becomes dark and stops interacting with the resonant laser. In this case, we also measured $g^{(2)}(\tau) = 1$. We therefore conclude that the observed photon bunching is due to the interaction with the QD as is fully explained and in accordance with the theoretical model.

Supplementary References

[1] S. Hughes, L. Ramunno, J. F. Young and J. E. Sipe. *Extrinsic Optical Scattering Loss in Photonic Crystal Waveguides: Role of Fabrication Disorder and Photon Group Velocity.* Phys. Rev. Lett. **94**, 033903 (2005).

[2] J. P. Hugonin, P. Lalanne, T. P. White and T. F. Krauss. *Coupling into slow-mode photonic crystal waveguides.* Opt. Lett. **32**, 2638–2640 (2007).

[3] A. Faraon, I. Fushman, D. Englund, N. Stoltz, P. Petroff and J. Vučković. *Dipole induced transparency in waveguide coupled photonic crystal cavities.* Opt. Express **16**, 12154–12162 (2008).

[4] I. J. Luxmoore, N. A. Wasley, A. J. Ramsay, A. C. T. Thijssen, R. Oulton, M. Hugues, S. Kasture, V. G. Achanta, A. M. Fox and M. S. Skolnick. *Interfacing spins in an InGaAs quantum dot to a semiconductor waveguide circuit using emitted photons.* Phys. Rev. Lett. **110**, 037402 (2013).

[5] M. Arcari, I. Söllner, A. Javadi, S. Lindskov Hansen, S. Mahmoodian, J. Liu, H. Thyrrestrup, E. H. Lee, J. D. Song, S. Stobbe and P. Lodahl. *Near-unity coupling efficiency of a quantum emitter to a photonic crystal waveguide.* Phys. Rev. Lett. **113**, 093603 (2014).

[6] J. Johansen, B. Julsgaard, S. Stobbe, J. M. Hvam and P. Lodahl. *Probing long-lived dark excitons in self-assembled quantum dots.* Phys. Rev. B **81**, 081304(R) (2010).

[7] A. Auffèves-Garnier, C. Simon, J.-M. Gérard and J.-P. Poizat. *Giant optical nonlinearity induced by a single two-level system interacting with a cavity in the Purcell regime.* Phys. Rev. A **75**, 053823 (2007).

[8] A. V. Kuhlmann, J. Houel, A. Ludwig, L. Greuter, D. Reuter, A. D. Wieck, M. Poggio and R. J. Warburton. *Charge noise and spin noise in a semiconductor quantum device.* Nat. Phys. **9**, 570–575 (2013).

[9] H. S. Nguyen, G. Sallen, M. Abbarchi, R. Ferreira, C. Voisin, P. Roussignol, G. Cassabois and C. Diederichs. *Photoneutralization and slow capture of carriers in quantum dots probed by resonant excitation spectroscopy.* Phys. Rev. B **87**, 115305 (2013).

[10] D. Englund, A. Faraon, I. Fushman, N. Stoltz, P. Petroff and J. Vučković. *Controlling cavity reflectivity with a single quantum dot.* Nature **450**, 857–861 (2007).

[11] K. Konthasinghe, J. Walker, M. Peiris, C. K. Shih, Y. Yu, M. F. Li, J. F. He, L. J. Wang, H. Q. Ni, Z. C. Niu and A. Muller. *Coherent versus incoherent light scattering from a quantum dot.* Phys. Rev. B **85**, 235315 (2012).

[12] C. Matthiesen, M. J. Stanley, M. Hugues, E. Clarke and M. Atatüre. *Full counting statistics of quantum dot resonance fluorescence.* Sci. Rep. **4**, 4911 (2014).

[13] A. Reinhard, T. Volz, M. Winger, A. Badolato, K. J. Hennessy, E. L. Hu and A. Imamoğlu. *Strongly correlated photons on a chip.* Nat. Photonics **6**, 93–96 (2012).

[14] A. Faraon, I. Fushman, D. Englund, N. Stoltz, P. Petroff and J. Vučković. *Coherent generation of non-classical light on a chip via photon-induced tunnelling and blockade.* Nat. Phys. **4**, 859–863 (2008).

[15] K. Hennessy, A. Badolato, M. Winger, D. Gerace, M. Atatüre, S. Gulde, S. Fält, E. L. Hu and A. Imamoğlu. *Quantum nature of a strongly coupled single quantum dot-cavity system.* Nature **445**, 896–899 (2007).

[16] J. R. Johansson, P. D. Nation and F. Nori. *QuTiP 2: A Python framework for the dynamics of open quantum systems.* Comp. Phys. Comm. **184**, 1234–1240 (2013).

[17] H. Zheng, D. Gauthier and H. Baranger. *Waveguide QED: Many-body bound-state effects in coherent and Fock-state scattering from a two-level system.* Phys. Rev. A **82**, 063816 (2010).

[18] D. Witthaut, M. D. Lukin and A. S. Sørensen. *Photon sorters and QND detectors using single photon emitters.* Europhys. Lett. **97**, 50007 (2012).

[19] T. C. Ralph, I. Söllner, S. Mahmoodian, A. G. White and P. Lodahl. *Photon sorting, efficient Bell measurements, and a deterministic controlled-Z gate using a passive two-level nonlinearity.* Phys. Rev. Lett. **114**, 173603 (2015).
